# Case‐Mix Adjustment of Multidimensional Patient‐Reported Outcomes to Assess Hospital Performance

**DOI:** 10.1002/hec.70139

**Published:** 2026-08-04

**Authors:** Nils Gutacker, Yuanyuan Gu

**Affiliations:** ^1^ Centre for Health Economics University of York Heslington UK; ^2^ Macquarie University Centre for the Health Economy Macquarie University Sydney Australia

**Keywords:** case‐mix adjustment, EQ‐5D, PROMs, provider performance

## Abstract

Multidimensional patient‐reported outcome measures (PROMs) are increasingly used to compare the performance of health care providers. Meaningful comparisons require the data to be adjusted for differences in case‐mix. The common approach in the literature is to aggregate PROM data into univariate summary scores prior to case‐mix adjustment. In this paper, we extend the work by McCarthy (2015, 2016) to develop a dimension‐specific adjustment methodology for performance assessment. This method permits to model the influence of patient characteristics separately for each dimension while retaining the maximum amount of information to inform different stakeholders. We conduct a simulation study to compare the performance of both methods in terms of their ability to identify provider performance, and to identify context‐specific factors that should guide the choice of methodology in applied research. We follow this up with a real‐world case study of knee replacement surgery in the English NHS. We find that the domain‐specific approach outperforms the summary score approach in most cases but the differences are small. We conclude that the choice of method ought to consider not just performance but also ease of implementation and retention of decision‐relevant granular information.

## 1 Introduction

There is increasing interest in using patient‐reported outcome measures (PROMs) to compare the performance of health care providers in terms of the improvements in health‐related quality of life (HRQoL) that they achieve for their patients (OECD Health Ministers 2017). Several health systems, most notably those in England, Wales, US (Medicare), Canada, Australia and Sweden collect PROM data routinely and at scale for a subset of their patient populations (Kendir et al. 2026). Such information may be useful for prospective patients who wish to choose a provider of care (Gutacker et al. 2016), for providers to monitor and evaluate quality improvement activities (Rolfson et al. 2016), or for payers to link reimbursement to health outcomes (Siciliani et al. 2026; Plate et al. 2024). Because patients are not randomly allocated to health care providers, differences in post‐treatment HRQoL may not simply reflect differences in provider effort, but also the mix of patients treated in terms of, for example, their age, gender or the presence of comorbid conditions (Iezzoni 2012).^1^ It is therefore necessary to adjust for imbalances in confounding factors through statistical modeling: a process known as “case‐mix adjustment” (CMA). However, the characteristics of PROMs data may require a different CMA approach from that commonly used for other outcomes such as mortality or readmission rates.

Most PROM instruments consist of multiple items and cover more than one HRQoL domain. For example, the EQ‐5D‐3L, one of the most widely used PROM instruments, asks respondents to indicate the degree of problems they experience (no/some/extreme) on five dimensions (mobility (MO), self‐care (SC), usual activities (UA), pain & discomfort (PD), and anxiety & depression (AD)) that, together, describe a health profile (Brooks 1996). These multidimensional data can be aggregated into summary scores (SS) using a set of weights. Most commonly, these scores are interpreted as “values” using weights derived from studies of patient or population preferences.

The current practice in many PROMs programmes is to apply CMA to PROM summary scores (Nuttall et al. 2015); thereby mirroring the procedure for univariate outcomes. The two main advantages of the SS approach are that (i) the resulting adjusted values are expressed on a cardinal scale and (ii) adjustment can be performed using simple regression models suitable for continuous outcomes, such as OLS. However, the SS approach has some disadvantages: First, it requires profile data to be converted to scores before the case‐mix adjustment is made. This means that the case‐mix adjustment is only valid for the particular set of weights that is used. There is no flexibility, short of re‐estimating the case‐mix adjusted results, to use other sets of weights, for example where local decision makers prefer to use different sets of weights more applicable to their local patient population. Secondly, the detailed health profile information is lost in this process as the profiles cannot be recovered from the predicted value. This may limit the usefulness of the performance data for quality improvement activities (Øvretveit et al. 2017), for example clinicians seeking ways to improve their care by examining their performance on the different health dimensions (Gutacker et al. 2013), or for patients who seek to understand how surgery affects different aspects of their lives (Gutacker and Street 2017). Finally, using *any* set of weights introduces an exogenous source of variance to profile data (Parkin et al. 2010), which may affect the statistical performance of the CMA.

McCarthy (2015, 2016) proposed a methodology to estimate treatment effects expressed as differences between two groups (e.g., a new treatment vs. standard care) in terms of SF‐6D profiles, which are subsequently aggregated into values.^2^ Following this line of thinking, we propose a *domain response* (DR) approach to hospital performance assessment in which EQ‐5D‐3L responses are first adjusted for patient characteristics within each health dimension and the resulting adjusted dimension responses are aggregated into health profiles that can be translated into values. This method is true to the nature of the EQ‐5D‐3L instrument as a descriptive system that is distinct from valuation. However, estimating and predicting multivariate categorical data may result in estimates that are more uncertain than those based on risk‐adjustment of single, continuous values. Thus there is a potential trade‐off between appropriateness and precision.

The aim of this study was to establish if and under which circumstances CMA using the DR approach performs as well as or better than adjustment using the SS approach. To our knowledge, there has not been a systematic comparison of these alternative CMA approaches for the purpose of health care provider performance assessments. We contribute to the literature by conducting a simulation study to compare both approaches head‐to‐head in terms of their ability to recover true provider performance under a set of assumptions about the data generating process. We also applied both methods to a case study of real‐world data taken from the English National Health Service (NHS). Our results suggest that the DR approach generates better estimates of provider performance than the SS approach in most realistic scenarios. This is particularly evident when there is a large number of case‐mix factors to adjust for, and case‐mix variation is large relative to performance variation. However, the difference in predictive ability between both approaches is typically small in absolute terms.

## 2 Two Approaches to Case‐Mix Adjustment

We follow common practice in provider performance assessment and assume that the adjusted performance estimate ${\hat{\bar{Y}}}_{j}$ for provider j=1,…,J is obtained by means of the following indirect standardization process (NHS England 2013):

(1) $${\hat{\bar{Y}}}_{j}=\frac{1}{N}\sum_{i=1}^{N}Y_{ij}+\frac{1}{N_{j}}\sum_{i=1}^{N_{j}}\left(Y_{ij}-{\hat{Y}}_{ij}\right)$$

where $Y_{ij}$ and ${\hat{Y}}_{ij}$ represent the observed and expected outcome for the ith patient at the jth provider respectively, $N_{j}$ the patient volume for the jth provider, and N the total patient volume of all J providers. The first term in the equation is the average outcome calculated over the full patient population and the second term is the hospital‐specific average difference between observed and expected outcomes in provider j’s patient population.^3^ Note that the first term is constant across all providers j=1,…,J and acts as a scaling parameter.

An important analytical task—and the overarching focus of this study—is to calculate an unbiased estimator ${\hat{Y}}_{ij}$. One possible approach is to estimate an OLS regression model of the effect of a vector of patient characteristics^4^ on observed EQ‐5D‐3L values, i.e.

(2) $$Y_{ij}=\alpha +\gamma \times X_{ij}+\varepsilon_{ij}$$

and then use the regression coefficients to predict ${\hat{Y}}_{ij}$.

Alternatively, analysts can estimate individual regression models for each of the five EQ‐5D‐3L dimensions and then use the predictions to generate health profiles and summary scores. Since the observed dimension responses are ordinal in nature, we propose to use ordered probit (or logit) models with the same set of regressors that are used in the SS regression.^5^ This modeling approach recognizes that the effect of the patient characteristic $X_{ij}$ may vary across EQ‐5D‐3L domains. The resulting coefficient estimates are used to predict the probabilities of reporting each response for each patient i. The probability of a given health profile can then be calculated as joint probabilities over the five dimensions. For simplicity, assume that dimension responses are uncorrelated,^6^ so that the probability of a patient with observed characteristic $X_{i}$ reporting no problems on any dimension is given by

(3) $$\begin{array}{cc} \mathrm{Pr}\left(PROFILE=11111|X_{i}\right) & =\mathrm{Pr}\left(MO=1|X_{i}\right)\times \mathrm{Pr}\left(SC=1|X_{i}\right)\\ & \;\times \mathrm{Pr}\left(UA=1|X_{i}\right)\times \mathrm{Pr}\left(PD=1|X_{i}\right)\\ & \;\times \mathrm{Pr}\left(AD=1|X_{i}\right) \end{array}$$

and similarly for all other health profiles.

The expected summary score for patient i can then be calculated as

(4) $${\hat{Y}}_{ij}=\sum_{h=1}^{H}\mathrm{Pr}\left(PROFILE_{h}|X_{i}\right)\times value\left(PROFILE_{h}\right)$$

i.e. the sum of the products of the probability of each EQ‐5D‐3L health profile h and its corresponding valuation. Table A1 in the Appendix provides a worked example.

## 3 Simulation Study

### 3.1 Monte Carlo Simulation and Methods Evaluation

We use Monte Carlo simulation to evaluate the performance of both CMA approaches under experimental conditions. We conduct nine sets of analyses: one base case and eight scenario analyses. For each analysis, we create a series of S=1,000 simulated data sets of patient‐level observations with complete EQ‐5D‐3L profiles and associated summary scores (“values”). The observed profile for each patient‐level observation is determined by three components that can be manipulated as part of the experiment: the hospital effect (“performance”), the effect of one or more patient characteristics (i.e., confounders), and random noise. Values for these components are drawn from random distributions and predictions are generated under specific assumptions, e.g. about the number of case‐mix factors or the set of weights used.

In each simulated data set, we create adjusted hospital performance estimates using the process outlined in Section 3.2. We then compare these adjusted estimates against the known true hospital performance levels and calculated two statistics: the root mean squared prediction error (RMSE) and the Spearman rank correlation index (ρ) between true and calculated performance estimates. We also record which of the two CMA approaches performs better on each metric. Results are averaged over all simulations.^7^

### 3.2 Data Generating Process

This section sets out how the simulated data are generated. Table 1 summarizes the assumptions about the data generating process (DGP) used in the base case. All analyses were conducted in Stata 18 (StataCorp LLC; College Station, TX). Software code to create the simulated data is provided as online Supporting Information S1.

**TABLE 1:** Assumptions about data generating process—base case.

Parameter	Description	Distribution	Values
J	Number of hospitals	Fixed	J = 100
$N_{j}$	Number of patients in hospital j	Uniform(a,a×10)	a = 30
M	Number of case‐mix characteristics	Fixed	M = 3
${\bar{X}}_{j,m}$	Proportion of patients in hospital j for which case‐mix factor m equals 1	Uniform(0, $u_{m}$) where $u_{m}$ follows Uniform(0,1)	
β	Effect of each case‐mix characteristic (on latent scale)	Uniform(−c,c)	c = 3
θ	Hospital performance (on latent scale)	Normal$\left(0,\tau^{2}\right)$	$\tau =\sqrt{0.5}$
ϵ	Random noise (on latent scale)	Normal$\left(0,r\times \tau^{2}\right)$	r = 1
$cov\left(\epsilon_{\mathrm{ijk}}^{UA},\epsilon_{\mathrm{ijk}}^{PD}\right)$	Correlation between domains	Fixed	0
	EQ‐5D‐3 L value set	Fixed	MVH‐A1^a^

^a^ This is automated through the user‐written command eq5d by Ramos Goni & Rivero Arias.

We first create a data set of j=1,…,100 providers. For each provider j we generate a performance component $\theta_{j}∼N\left(0,\tau^{2}\right)$, the number of patients the provider treats $N_{j}∼U\left(a,b\right)$, and the proportion of patients with the m=1,…,M case‐mix characteristics of interest ${\bar{X}}_{j,m}∼U\left(0,u_{m}\right)$ by drawing random values from the respective probability distributions. We assume that there is only a single performance component that affects all five latent HRQoL dimensions k=1,…,5. This ensures that providers can be ranked on $\theta_{j}$, assuming no ties.

We then expand the data set to create individual patient records that are nested within providers. For each patient $i=1,\text{…},N_{j}$ in provider j we generate a set of m binary case‐mix characteristics $X_{ij,m}$ with $\mathrm{Pr}\left(X_{ij,m}=1\right)={\bar{X}}_{j,m}$. Because ${\bar{X}}_{j,m}$ varies across providers, this creates differences in providers' case‐mix. We also create random noise in HRQoL as $\epsilon_{\mathrm{ijk}}∼N\left(0,\sigma_{k}^{2}\right)$ with $cov\left(\epsilon_{\mathrm{ijk}},\epsilon_{ijk^{\prime }}\right)=0$ for all $k\neq k^{\prime }$. We relax the assumptions of uncorrelated responses across HRQoL domains as well as normal, homoscedastic error terms in scenario analyses.

Patients' HRQoL with respect to each of the five dimensions of the EQ‐5D‐3L is represented by a latent scale model (Long 1997; Gutacker et al. 2013), in which

(5) $$y_{\mathrm{ijk}}^{*}=\theta_{j}+X_{ij}\times \beta_{k}+\epsilon_{\mathrm{ijk}}$$

where $y_{\mathrm{ijk}}^{*}$ is patient i’s level of latent HRQoL on dimension k (e.g., mobility) and the vector $\beta_{k}∼U\left[-c,c\right]$ denotes the effect of the matrix of binary case‐mix characteristic $X_{ij}$ on latent HRQoL. These latent scores are then translated into observed responses on the ordinal scale of the EQ‐5D‐3L instrument by means of the standard threshold model, so that

(6) $$y_{\mathrm{ijk}}=\left\{\begin{array}{c} 3,\;if\;y_{\mathrm{ijk}}^{*}\leq \kappa_{1k}\\ 2,\;if\;\kappa_{1k}<y_{\mathrm{ijk}}^{*}\leq \kappa_{2k}\\ 1,\;if\;y_{\mathrm{ijk}}^{*}>\kappa_{2k} \end{array}\right.$$

where the threshold parameters κ are chosen according to percentiles of the distribution of the latent variable $y_{\mathrm{ijk}}^{*}$ so that pre‐specified proportions of patients report either no, some or extreme problems on a given dimension as shown in Table 2.^8^ In the last step, dimension responses are combined into EQ‐5D‐3L health profiles, which are scored using a value set.

**TABLE 2:** Simulated proportion of dimension responses.

Dimension	No problems (= 1)	Some problems (= 2)	Extreme problems (= 3)
Mobility (MO)	70%	18%	12%
Self‐care (SC)	80%	14%	6%
Usual activities (UA)	50%	30%	20%
Pain & Discomfort (PD)	65%	25%	10%
Anxiety & Depression (AD)	55%	25%	20%

### 3.3 Benchmark

To measure the ability of different CMA approaches to recover estimates of true performance, we require hospital‐specific benchmarks against which both methods can be compared. The parameter $\theta_{j}$ cannot be used directly as a benchmark value for ${\hat{\bar{Y}}}_{j}$ because the data generating process involves a non‐linear transformation of a linear predictor, which includes the performance component $\theta_{j}$ and some case‐mix influence, onto the ordinal scales of the EQ‐5D‐3L dimensions. Instead, we create health profile data following the same process set out above but fixing case‐mix to be equal across providers.

For each simulated patient i in provider j we calculate their responses on each of the five EQ‐5D‐3L dimensions if they had characteristic $X_{ij}=\bar{\bar{X}}$, i.e. all patients have their characteristic set to the same value that equals the average value in the patient population (and, hence, $\mathrm{var}\left(X_{ij}\right)=0$). So

(7) $$y_{\mathrm{ijk}}^{*}=\theta_{j}+\bar{\bar{X}}\times \beta_{k}+\epsilon_{\mathrm{ijk}}$$

where $\bar{\bar{X}}$ is a constant and all systematic variation across hospitals arises because of $\theta_{j}$. These values are then transformed into 3‐level responses using the approach set out in Equation (6) with the same set of values for $\kappa_{1}$ and $\kappa_{2}$ as before. The resulting benchmark value for each hospital is denoted as $Y_{j}^{\mathrm{TRUE}}$. Figure 1 shows average EQ‐5D‐3L values by hospital with associated 95% confidence intervals when scores are calculated allowing for case‐mix variation (Equation 5) or not (Equation 7).

**FIGURE 1:**
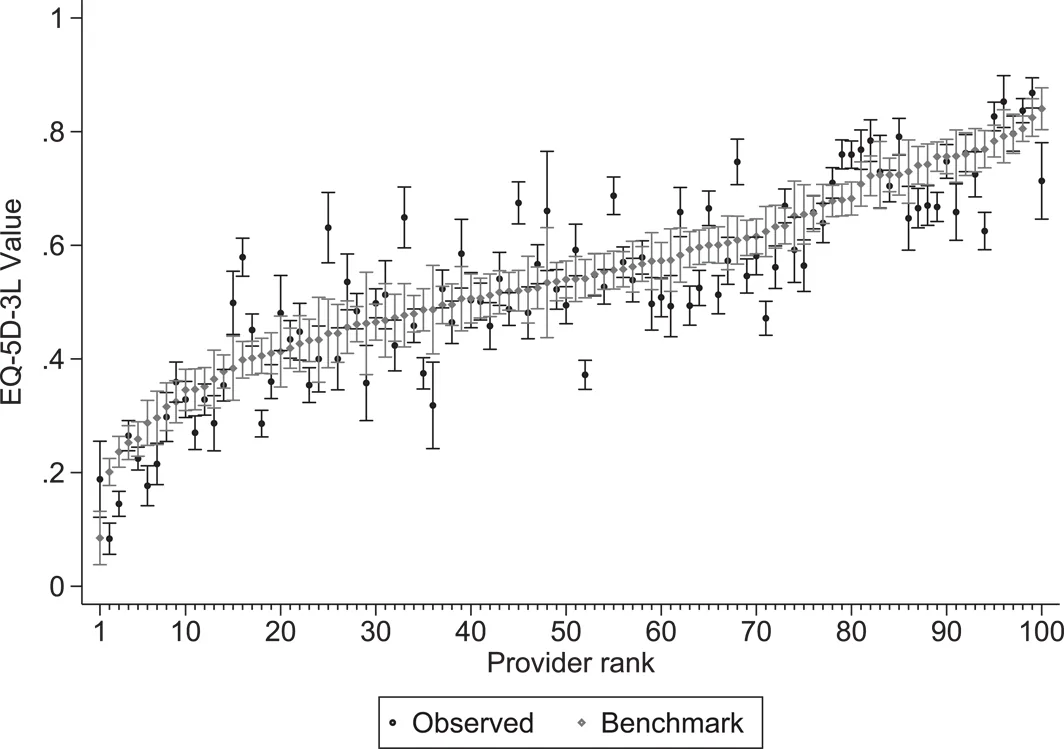
Simulated average EQ‐5D‐3L values by hospital with and without case‐mix variation (base case).

## 4 Results

### 4.1 Simulation

Table 3 presents the main findings of the simulation study. In our base case analysis, the DR approach creates provider level performance estimates that are closer to the known benchmark as indicated by, on average, lower RMSE (0.043 vs. 0.053) (see also Figure 2) and higher Spearman rank correlation coefficients (ρ: 0.979 vs. 0.949). However, the DR approach does not perform favorably in all simulations and parameter constellations. For example, in 8% of simulations the SS approach had lower RMSE than the DR approach.

**TABLE 3:** Simulation results—base case and alternative scenarios.

	Successful	RMSE			Spearman correlation (ρ)		
Scenario	simulations ^a^	DR	SS	DR > SS	DR	SS	DR > SS
Base case	1000	0.043	0.053	92%	0.979	0.949	98%
Scenario 1: Number of case‐mix factors							
1	1000	0.041	0.034	1%	0.986	0.987	34%
2	1000	0.043	0.047	55%	0.982	0.971	81%
3 ^b^	0.043	0.054	93%	0.979	0.948	99%	
4	1000	0.041	0.057	99%	0.977	0.925	100%
5	1000	0.040	0.058	100%	0.976	0.909	100%
Scenario 2: Range of β values sampled (c in U(−c,c))							
1	1000	0.044	0.036	4%	0.987	0.986	70%
2	1000	0.043	0.047	70%	−0.983	−0.971	96%
3 ^b^	1000	0.042	0.053	94%	0.979	0.947	99%
4	999	0.042	0.056	96%	−0.975	−0.925	99%
5	998	0.041	0.059	98%	0.972	0.898	99%
Scenario 3: Ratio of var(θ) to var(ϵ)							
0.2	1000	0.032	0.050	100%	0.846	0.611	100%
0.6	1000	0.036	0.051	98%	0.967	0.895	100%
1.0 ^b^	1000	0.042	0.054	92%	0.979	0.948	98%
1.4	1000	0.049	0.056	76%	0.982	0.965	95%
1.8	1000	0.058	0.058	48%	0.981	0.972	89%
Scenario 4: EQ‐5D value set^c^							
MVH‐A1^b^	1000	0.043	0.053	92%	0.979	98%	0.949
Equal weights	1000	0.021	0.026	99%	0.982	98%	0.962
Scenario 5: Minimum hospital volume a							
10	1000	0.060	0.069	90%	0.963	0.933	98%
20	1000	0.047	0.058	91%	0.975	0.944	98%
30^b^	1000	0.043	0.053	92%	0.979	0.949	98%
40	1000	0.039	0.051	92%	0.981	0.951	99%
50	1000	0.038	0.050	92%	0.982	0.952	98%
Scenario 6: Ignoring the correlation between UA and PD $\left(cov\left(\epsilon_{k}^{UA},\epsilon_{k^{\prime }}^{PD}\right)\right)$ ^d^							
0.0 ^b^	1000	0.043	0.053	92%	0.979	0.949	98%
0.1	1000	0.043	0.053	91%	0.979	0.949	98%
0.2	1000	0.042	0.053	93%	0.979	0.947	99%
0.3	1000	0.043	0.053	90%	0.978	0.948	98%
0.4	1000	0.043	0.053	87%	0.978	0.949	97%
0.5	1000	0.044	0.054	86%	0.977	0.946	98%
Scenario 7: Heteroscedastic errors with $\mathrm{var}\left(\epsilon_{k}\right)=\sigma_{k}^{2}$ if $X_{1}=0$ and $\mathrm{var}\left(\epsilon_{k}\right)=2\sigma_{k}^{2}$ if $X_{1}=1$							
No (homoscedastic) ^b^	1000	0.043	0.054	93%	0.979	0.948	99%
Yes	1000	0.046	0.057	92%	0.978	0.946	98%
Scenario 8: Logistic error distribution with scale parameter s (mean = 0, var = $s^{2}\pi^{2}/3$)							
0.6	1000	0.037	0.045	92%	0.976	0.951	98%
1	1000	0.030	0.034	90%	0.963	0.948	94%
1.4	1000	0.026	0.028	85%	0.942	0.934	85%

^a^ Total number of scenarios conducted: 1000.

^b^ Base case.

^c^ MVH‐A1 is the preferred UK value set (Dolan 1997). The “equal weight” value set assigns a constant decrement of 0.1 to each increase in response level (e.g., from *no* to *some* problems, or from *some* to *extreme* problems) on each EQ‐5D dimension.

^d^ The DR model was fitted using independent ordered probit models, thus ignoring the correlation between the two domains UA and PD in estimation and prediction.

**FIGURE 2:**
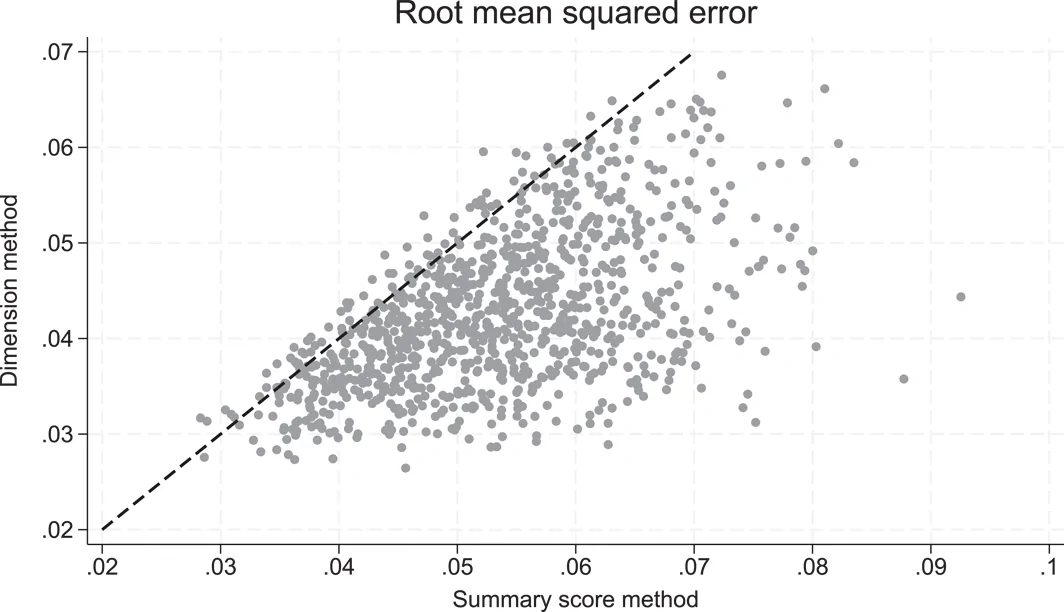
Root mean squared error (RMSE) across 1000 simulations of base case scenario. Every dot denotes one of S =1,000 simulations. Dashed line shows 45 degree line of equality.

To further explore the determinants of the relative performance of both methods, we conducted several scenario analyses. We first changed the relative amount of variation due to case‐mix and other factors (i.e., var(X×β) and var(θ)+var(ϵ)). We did so by varying the number of case‐mix factors m (Scenario 1) and the impact of case‐mix factors on latent HRQoL i.e. the β parameters (Scenario 2). These analyses reveal that the relative performance of the DR approach increases as the share of total variation due to case‐mix increases. Only when there are very few CM factors or when they have little impact on HRQoL the SS approach performs better than the DR approach.

We also explored whether the results are sensitive to the signal‐to‐noise ratio between true performance variation, var(θ), and random variation, var(ϵ), holding the role of case‐mix variation constant (Scenario 3). As before, we find that the DR approach tends to generate hospital estimates with less prediction error and whose rankings are more closely aligned with those of the benchmark. Differences diminish as the ratio of var(θ) to var(ϵ) increases. For very high ratios (here: 1.8 and above) the average RMSE is found to be lower under the SS approach. However, such ratios are several times larger^9^ than those commonly found in the performance assessment literature in health care (e.g., Gutacker et al. 2018), suggesting that this finding is of limited practical concern.

We do not find evidence to support the hypothesis that the choice of EQ‐5D‐3L value set impacts on the relative performance of the two methods (Scenario 4). We find some evidence that the number of patients per provider, and thus the overall sample size over a fixed number of providers, affects relative performance (Scenario 5). This is likely to reflect the additional statistical uncertainty resulting from having to estimate and predict five instead of one outcomes, which is more pronounced in small samples.

We explored how correlation across EQ‐5D‐3L domains, if ignored, impacts the performance of each method (Scenario 6). While the change in RMSE under both alternative approaches is small, we find that the SS approach is increasingly being favored as the correlation between domains increases. These results can be interpreted in terms of the extent to which EQ‐5D‐3L domains provide non‐overlapping information. Where inter‐domain correlation is low, the multidimensional response profile contains distinct signals on different aspects of health, and a DR approach may therefore offer an informational advantage over adjustment based on an aggregated SS. By contrast, as correlation across domains increases, the profile approaches a more unidimensional construct, such that each individual domain contributes less additional information conditional on the others. Under these conditions, the relative benefit of modeling domains separately diminishes and the SS approach becomes more competitive.

Finally, we explored how different error distributions affect the performance. We simulated heteroscedastic error terms where the variance of the error term doubles in the presence of the binary variable X1, i.e. $\mathrm{var}\left(\epsilon_{k}\right)=\left(1+X_{1}\right)*\sigma_{k}^{2}$ (Scenario 7). Our simulations found the RMSE to increase for both the DR and SS approach, yet the performance difference remains mostly unchanged. We also simulated error terms that come from a logistic distribution and found the DR approach to outperform the SS approach despite the misspecification of the Probit regression model, although this performance difference reduced as the scale parameter, and thus the variance of the distribution, increased (Scenario 8).

### 4.2 Real‐World Case Study

To assess the practical implications of choosing one adjustment method over another, we applied both to publicly available data on N = 34,640 patients receiving NHS‐funded knee replacement surgery in J = 213 hospitals in England between April 2019 and March 2020.^10^ Hospitals are compared in terms of average EQ‐5D‐3L summary scores measured at 6 months after surgery. The following CM factors were adjusted for in line with official procedure (NHS England 2013): patients' age (in 10‐year bands), sex, presence of up to 12 long‐standing conditions (e.g., diabetes, hypertension, lung disease), symptom duration, pre‐operative EQ‐5D‐3L health profile, and whether the patient reported to live alone or to require assistance with filling in the questionnaire.

Patients reported 128 different post‐operative EQ‐5D‐3L profiles, or approximately 53% of all profiles that can be generated by this instrument. The frequency distribution was highly skewed with 67% of patients clustered in just five profiles.^11^ There were few patients that reported extreme problems on any domain (MO = 0.1%, SC = 0.4%, UA = 2.9%, PD = 4.3%, AD = 2.2%), and very few that reported extreme problems on more than one domain (1.8%). The average pre‐ and post‐operative utility scores were 0.43 (SD = 0.31) and 0.76 (SD = 0.24), respectively, and most patients (83.5%) reported an improvement in utility scores following surgery.

Hospitals' true performance levels are not observed and therefore cannot serve as benchmarks. Instead, we follow Gutacker et al. (2020) and compare for each hospital their performance status (i.e., whether they were deemed to perform well, as expected, or poorly) under the two adjustment procedures. Substantial differences in classification under both methods would give cause for concern, although they could not be used in isolation to determine which method is preferable. Performance statements are derived from funnel plots that account for sampling uncertainty (Spiegelhalter 2005). Providers are deemed to perform well if their mean adjusted SS, ${\hat{\bar{Y}}}_{j}$, is at least two standard deviations above the sample average (solid line) as indicated by the upper control limit (dashed line). Similarly, providers are considered poor performers if their average adjusted outcomes is more than two standard deviations below the sample average.

Table 4 shows a cross‐tabulation of performance judgments under both CM adjustments.^12^ The resulting provider performance estimates are highly correlated (Spearman ρ = 0.997) and identify the same high‐ and low‐performing hospitals, with only one divergence. A similar result is obtained when comparing hospital ranks rather than performance categories (Figure 3). We only observe small changes in rank estimate, and these are concentrated on the middle of the rank distribution, where rank uncertainty tends to be large and provider estimates are poorly differentiated.^13^ These results suggest that both methods perform similarly and would lead to very similar conclusions in this particular context.

**TABLE 4:** Cross‐tabulation of performance judgments under both approaches.

	DR approach			
SS approach	Good	As expected	Poor	Total
Good	18	0	0	18
As expected	0	176	0	176
Poor	0	1	18	19
Total	18	177	18	213

*Note:* Every dot denotes one hospital (*N* = 213).

**FIGURE 3:**
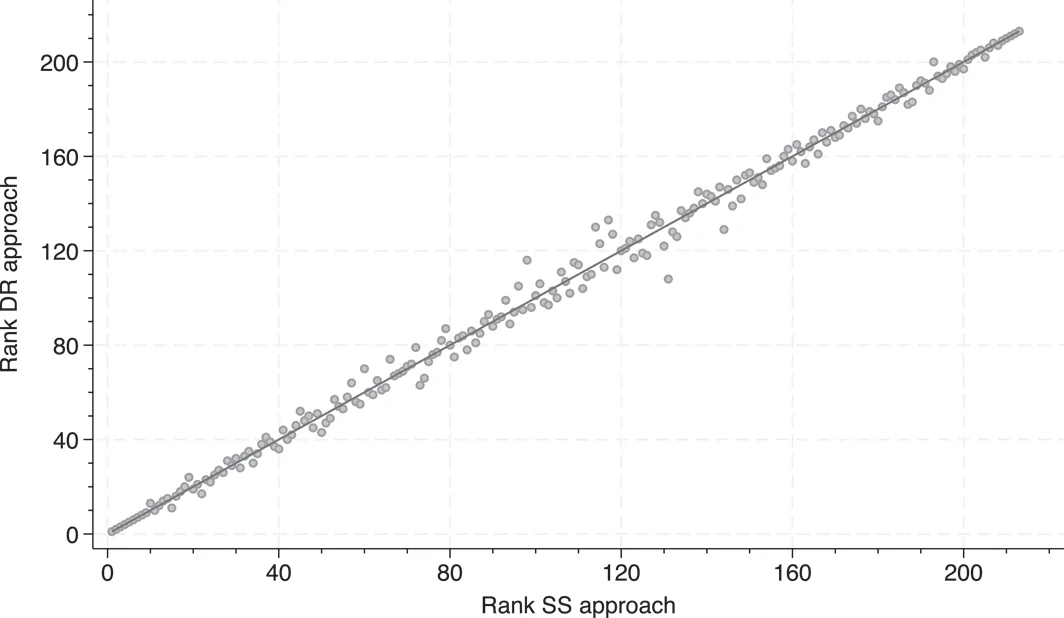
Hospital league table rankings under both approaches. Every dot denotes one hospital (N=213).

## 5 Conclusions

Multi‐attribute utility instruments ask patients to report their HRQoL along multiple domains and are now being collected routinely in many health systems (Kendir et al. 2026; Rolfson et al. 2016; Ernstsson et al. 2020). These data can be aggregated into single‐number summary scores but CMA can be applied before or after aggregation. Our simulation study has shown that the DR approach, which first adjusts health profiles and then aggregates these into SS, has lower prediction error and generates estimates that are more closely correlated with true performance than a commonly used CMA approach that adjusts SS directly. This is particularly evident when the number of patient characteristics or the effect of these characteristics on HRQoL is large. However, differences in predictive ability between methods appear to be small overall and of limited practical importance. This is also illustrated through a real‐world case study of knee replacement surgery in the English NHS in which both methods generate nearly identical classifications of which hospitals perform well, poorly or as expected. Based on our simulation study, we expect to find similar results in other health system applications with comparable caseloads.

The evidence presented in this study does not suggest that those charged with conducting performance assessments ought to switch methods because of concerns over potential misclassification bias. However, the DR approach offers greater flexibility than the SS approach that may be valuable in its own right if it supports patient choice or quality improvement efforts. Prospective patients may value different aspects of recovery differently, and provider comparisons based on domain‐specific information may therefore be more informative than comparisons based solely on an aggregated summary score (Gutacker et al. 2013). If a summary score is sought, the DR approach permits users of performance information to implement their own health state valuations rather than rely on “pre‐canned” valuations that may lead to misinformation (Gutacker et al. 2020).^14^ Furthermore, because the DR approach retains the health profile information it allows those charged with quality assurance to establish in which HRQoL domains their organization performs better or worse than their peers, which may guide quality improvement initiatives. For example, shortfalls on the pain & discomfort domain are likely to necessitate a different clinical response than higher than expected rates of problems with anxiety & depression; actionable information that is lost under the SS approach.

Our study has three important limitations. First, our simulation study assumes a particular DGP in which case‐mix factors have a linear effect on latent health and therefore a non‐linear effect on the probability of reporting no, some or extreme problems. This is also the assumption underpinning the ordered probit model that was used to implement the DR approach. Conversely, the OLS model used in the SS approach assumes, in effect, that case‐mix factors have a linear effect on the probability of reporting a particular response and therefore a linear effect on values. The assumed linear effect on latent health may be reasonable in many clinical applications. However, it is possible that the way in which the DGP was specified may have favored the DR approach. Second, because we fixed the proportion of simulated patients reporting no, some or extreme problems in each domain at the outset, we avoided issues with rare responses in categorical regressions analysis that can lead to unstable or biased estimates in small samples (King and Zeng 2001). This issue is likely to be amplified in PROMs with more response levels per dimension such as the EQ‐5D‐5L (Herdman et al. 2011). Because the DR approach, as implemented here, analyses each domain individually, any bias due to rare responses is likely to differ across domains, which may make it more difficult to detect. Future research should explore how the DR approach performs when some response categories are rarely used. Finally, we did not explore the impact of correlation amongst more than two HRQoL domains. We caution that correlation, if ignored, can impact the predictive ability of the DR approach as evidenced by our simulations and that correlation among more than two dimensions is likely to amplify this issue. Suitable methods to account for such correlation exist and are readily available in modern statistical software packages.

In conclusion, our findings suggest that the DR approach has merit as a tool for CMA of HRQoL data. However, further in‐depth exploration of its behaviors and potential advantages over the SS approach in the context of different DGPs are required before it can be recommended for use in national PROM programmes.

## Conflicts of Interest

The authors declare no conflicts of interest.

## Supporting information


Supporting Information S1

## Figures and Tables

**FIGURE A1:**
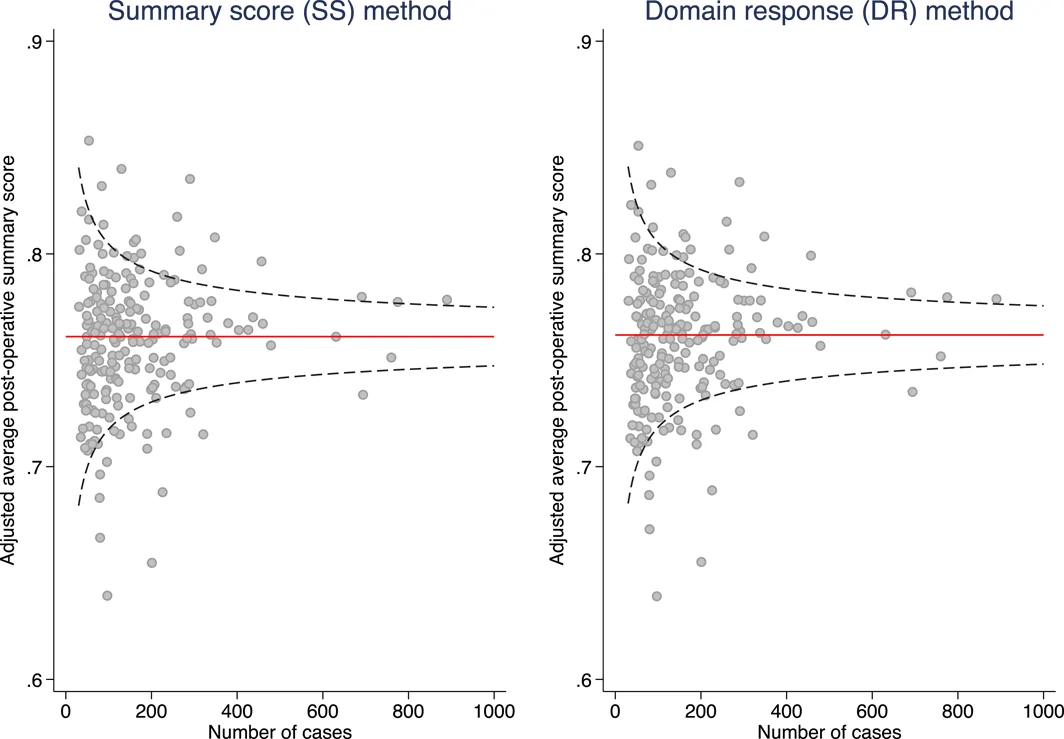
Funnel plots of hospital performance estimates based on HRQoL after knee replacement surgery in England (financial year 2019/20).

## Data Availability

The data that supports the findings of this study are available in the supplementary material of this article.

## Endnotes

^1^
Performance comparisons may also wish to control for provider characteristics that are out of providers' control such as whether they operate in an urban or rural location. These provider characteristics are not the focus of this paper although the proposed methodology would extend to this scenario as well.

^2^
See also a nascent literature on response mapping of HRQoL instruments that follows a similar logic; e.g. Gray et al. (2006).

^3^
Another popular method uses the ratio of observed and expected outcomes, which is calculated by a scaling parameter to recover the performance estimate. This method is usually employed with binary outcomes such as mortality within 30 days. In contrast, the method in (1) was developed by NHS England (2013) for use with continuous outcome variables such as EQ‐5D‐3L values and has been shown to result in more stable performance estimates.

^4^
More sophisticated modelling approaches such as two‐part models or Tobit regression are not commonly used in this application setting and are therefore not considered here.

^5^
Ordered probit models have the advantage that they can be extended more easily to multivariate regression models that allow for covariance in error terms across EQ‐5D‐3L dimensions.

^6^
The formula in (3) can easily be extended to allow for correlation by including appropriate terms for conditional probabilities.

^7^
Very occasionally, the regression analysis underpinning the DR approach did not converge. In this case, results for both methods were discarded using list wise deletion to ensure a consistent set of simulation results to average over.

^8^
Note that this way of creating dimension responses means that any changes to the model parameters (i.e., β, θ or ϵ) will affect the latent variable and its variance but not the distribution of observed responses. Since the proportions of observed responses are fixed, any changes to the latent variable will result in changes to the thresholds κ instead.

^9^
A ratio of 1.8 implies a variance partition coefficient of $\frac{\mathrm{var}\left(\theta \right)}{\mathrm{var}\left(\theta \right)+\mathrm{var}\left(\epsilon \right)}=\frac{1.8}{1.8+1}=64\%$, i.e. two thirds of unexplained variation in outcomes would be due to provider performance differences.

^10^
We only include patients who reported their HRQoL before and 6 months after surgery, and who had complete information on personal and medical characteristics used in the CM adjustment procedure.

^11^
These are: 11,111 (31.3%), 21,221 (12.2%), 11,121 (12.0%), 11,221 (6.5%), and 21,222 (4.8%).

^12^
See Appendix Figure A1 for funnel plots of hospital average scores derived under both approaches.

^13^
For an in‐depth discussion of the statistical challenges surrounding league tables and rank estimation see Goldstein and Spiegelhalter (1998).

^14^
Methods for the derivation of individual‐specific utility functions have been proposed and may be suitable for use within the clinical contact, e.g. as part of shared‐decision making involving referral decisions (Schneider, Devlin, et al. 2024b; Schneider, Blankart, et al. 2024a).

## Appendix

**TABLE A1:** Worked example: Probability of a given health profile and associated value.

Health Profile	Pr(MO)	Pr(SC)	Pr(UA)	Pr(PD)	Pr(AD)	Joint Probability	Value	Product
11111	0.990	0.811	0.481	0.303	0.044	0.005	1.000	0.005
11112	0.990	0.811	0.481	0.303	0.531	0.062	0.848	0.053
11113	0.990	0.811	0.481	0.303	0.425	0.050	0.414	0.021
11121	0.990	0.811	0.481	0.486	0.044	0.008	0.796	0.007
⋮								
33332	0.001	0.027	0.213	0.211	0.531	5$e^{-\;7}$	−0.429	−2$e^{-\;7}$
33333	0.001	0.027	0.213	0.211	0.425	4$e^{-\;7}$	−0.594	−2$e^{-\;7}$
							Total	0.410

*Note:* The joint probability is calculated as the product of all domain‐specific probabilities, assuming no correlation across domains. Value denotes the MVH‐A1 tariff (Dolan 1997) for the EQ‐5D‐3L health profile. Product gives the expected utility score by multiplying the joint probability with the health state value.
